# Special Issue “Centenarians—A Model to Study the Molecular Basis of Lifespan and Healthspan 2.0”

**DOI:** 10.3390/ijms241713180

**Published:** 2023-08-24

**Authors:** Calogero Caruso, Annibale Alessandro Puca

**Affiliations:** 1 Laboratory of Immunopathology and Immunosenescence, Department of Biomedicine, Neuroscience and Advanced Diagnostics, University of Palermo, 90134 Palermo, Italy; 2 Department of Medicine, Surgery and Dentistry “Scuola Medica Salernitana”, University of Salerno, 84081 Fisciano, Italy; apuca@unisa.it; 3 Cardiovascular Research Unit, IRCCS MultiMedica, 20138 Milan, Italy

## 1. State of the Art

The global population is experiencing an increase in ageing and life expectancy. The substantial increase in life expectancy, accompanied by the ensuing ageing of the population, stands as a remarkable human achievement. However, it also presents a formidable challenge confronting Western societies, given that ageing is intricately linked to heightened vulnerability to a spectrum of disease, including cardiovascular diseases (CVDs), cancer, type 2 diabetes, and Alzheimer’s disease (AD). The significance of ageing and longevity research in addressing the medical, economic, and societal challenges stemming from the rise in nonautonomous older individuals afflicted with debilitating ailments and diseases is intrinsically tied to the remarkable surge in the older population within the Western world [1]. In light of this, comprehending the mechanisms underpinning successful ageing and longevity emerges as an imperative endeavor. Such understanding is pivotal not only to embrace the positive dimensions of longevity, but also to counteract the adverse facets associated with ageing. As societies grapple with the complexities of an ageing demographic, a comprehensive grasp of the determinants of healthy ageing and the promotion of successful ageing is integral to devising strategies that mitigate the burdens posed by age-associated diseases [2].

In this context, delving into models of healthy ageing and extreme longevity assumes paramount significance. Among the spectrum of long-lived individuals (LLIs, >90), specific attention is directed towards centenarians (≥100 years), semi-supercentenarians (≥105 years), and supercentenarians (≥110 years); subjects from these categories are all under rigorous investigation. Among these, centenarians and even more so those who achieve an even longer life emerge as prime examples of successful aging. They have remarkably evaded infectious diseases in the pre-antibiotic era and withstood age-associated diseases, thereby serving as a beacon of positive biology [3,4]. Even though the hallmarks of ageing are relatively well understood [2], the genetic and epigenetic factors of longevity warrant heightened attention within the realm of ageing research.

A few years ago, we edited a Special Issue (S.I.) titled “Centenarians—a Model to Study the Molecular Basis of Lifespan and Healthspan”. The research and review articles featured in this Special Issue, titled “Centenarians—a Model to Study the Molecular Basis of Lifespan and Healthspan 2.0”, integrate and expand on that which was previously published [5]. The studies are centered on this paramount subject and align with the vital “positive biology” paradigm. This approach is designed to delve into the biological mechanisms that underlie healthy ageing, explored through either in vivo experiments in models or observational studies.

## 2. Genetics

The notion of longevity transcends mere genetic factors. A few years ago, this message echoed across global headlines, spurred on by a comprehensive study that meticulously dissected the genealogical histories of a staggering 400 million individuals. This remarkable endeavor included generational data encompassing birth and death dates, geographical origins, and intricate family connections. The study, coupled with another released concurrently, seemingly underscored the minimal role genes play in the attainment of longevity [6,7]. Nevertheless, it is crucial to note that these exhaustive investigations predominantly assessed the genetic impact on lifespan rather than longevity itself [8]. The focus of both papers rested on the limited predictability of future longevity based on the lifespans achieved by predecessors or parents. It is worth highlighting that these studies were grounded in age data that likely mirrored the average lifespan within the population, encompassing only a very small fraction of centenarians (less than 1 in 5000). This contextual understanding explains the observed low heritability associated with the trait of longevity. On a contrasting note, several years ago, Perls et al. [9,10] definitively demonstrated a robust heritability of the exceptional longevity phenotype. Notably, this heritability escalated even further with the advancing ages of the study participants [11].

In their review published in the S.I., Caruso et al. [11] extensively examined numerous studies that look at the genetics of longevity. The findings highlight a notable pattern: despite considerable efforts and advancements in technology, only two genes, APOE and FOXO3A, which are implicated in shielding against cardiovascular diseases, have demonstrated consistent associations with longevity across the majority of investigations [12]. This trend is attributed to the dynamic nature of genetic factors influencing longevity, intricately interwoven with the unique environmental history of specific populations. Notably, genes that are specific to particular populations appear to wield a more substantial influence on attaining longevity compared to those shared among diverse populations. Consequently, it is expected that the replication of associations from genome-wide association studies (GWAS) with common variants tied to longevity remains scarce due to the pooling of heterogeneous populations in these studies. A promising alternative avenue involves the study of families with a long-lived history. This approach is emerging as a valuable resource for unearthing protective genetic alleles and their associated biological signatures, which contribute to healthy ageing traits and exceptional longevity. By focusing on such familial lineages, researchers can potentially uncover more nuanced and population-specific insights into the genetic underpinnings of longevity [13].

In this S.I., a comprehensive effort was performed by Bae et al. [14] to conduct a GWAS focused on human extreme longevity (EL), characterized by surpassing the 99th percentile of survival. To achieve this, they synthesized data from four distinct centenarian studies [15], including 234 new EL cases, amalgamating a substantial dataset encompassing 2304 EL cases and 5879 controls. Through rigorous analysis, a locus within CDKN2B-AS1 (rs6475609, *p* = 7.13 × 10^−8^) emerged, presenting a noteworthy link to genome-wide significance. Additionally, four other loci exhibited suggestive levels of significance. Intriguingly, amidst these discoveries, a novel rare variant (rs145265196) on chromosome 11 emerged. This variant showed significantly higher longevity allele frequencies within Ashkenazi Jewish and Southern Italian ancestry cases when compared to those of other European ancestries. This underscores the diverse genetic underpinnings that might contribute to EL across distinct population groups (see above). Furthermore, a compelling facet of the study encompassed a correlation between EL-associated single-nucleotide polymorphisms and serum proteins. In particular, the authors evaluated the association between these rare longevity variants and the abundance of some circulating proteins that protect centenarians from cancer, AD, mitochondrial fission, and neutrophils activation. This proactive linkage sought to establish connections between the genetic findings and potential biological mechanisms associated with EL, thereby providing deeper insights into the genetic regulation underpinning longevity. Notably, the outcomes of the proteomic analyses hinted at the potential molecular pathways involved in promoting longevity. They suggested that significant genetic variants tied to longevity either provided EL cases with more youthful molecular profiles when compared to controls or offered a certain level of safeguarding against various diseases, including the progression of conditions such as AD. This interplay between genetics and proteomics illuminates the intricate relationship between the genome and the mechanisms that govern healthy ageing and resilience to age-related ailments.

To conclude the genetics section, it is noteworthy to highlight a recent study that, as discussed by Caruso et al. [11], conducted whole genome sequencing on 81 Italian semi- and supercentenarians [16] that, as previously stated, are more select groups than centenarians. The findings were subsequently juxtaposed with a cohort of geographically matched healthy individuals. The employed methods also facilitated the examination of somatic mutations. The outcomes unveiled a distinctive genetic makeup associated with EL, potentially attributed to an efficient DNA repair mechanism. This notion was corroborated by the notably low accumulation of somatic mutations, a characteristic comparable to that observed in younger control groups. This reduced load of somatic mutations could have contributed to shielding these individuals from CVDs.

## 3. Proteodynamics

The primary objective of the review authored by Frankowska et al. [17], and published in this S.I., was to explore the role of proteins in the context of longevity. The authors had previously demonstrated the intriguing observation that at least one intracellular proteolytic system exhibited comparable abundance in the peripheral blood lymphocytes of centenarians as that in the same cell type of young individuals [18]. Remarkably, cells from the older population exhibited a significant decline in this aspect when compared to both the young and centenarian cohorts. Amidst the limited available literature, the review embarked on a quest to address a fundamental question: how do distinct cell types of LLIs, ranging from nonagenarians to (semi-)supercentenarians, manage to uphold the integrity and quantity of their structural and functional proteins? Specifically, the inquiry focused on whether a more robust proteodynamic process contributes to longevity. The underlying hypothesis was that certain factors governing the maintenance of cellular proteomes in centenarians might remain at a “young” level, outperforming those found in the average older population. The exploration included a comprehensive analysis of various aspects of cellular protein maintenance, including the quality of transcribed DNA, its epigenetic modifications, the accuracy and extent of transcription for both mRNA and noncoding RNAs, the intricacies of the translation process, posttranslational modifications leading to the maturation and functionalization of nascent proteins, and the multifaceted process of eliminating misfolded, aggregated, and dysfunctional proteins through autophagy. The assessment also extended to the state of mitochondria, particularly their capacity for ATP production, which is vital for protein synthesis and maintenance. The findings yielded noteworthy insights. With the exception of chaperone function and mitochondrial status, almost all the examined aspects demonstrated enhanced performance in centenarians relative to the average older population. In fact, a substantial proportion of these aspects approached the levels or activities witnessed in the cells of young individuals. This nuanced investigation into the proteodynamic landscape of longevous individuals offers a multifaceted view of how various facets of cellular protein maintenance contribute to the remarkable phenomenon of longevity.

## 4. Models

The homozygous genotype of the Longevity-Associated Variant (LAV) within Bactericidal/Permeability-Increasing Fold-Containing Family B member 4 (BPIFB4) is enriched in the LLIs of three independent populations. The BPIFB4 protein, implicated in activating homeostatic processes such as adaptive stress response and proteostasis, is found at high levels in the serum of LLIs, and the LAV-BPIFB4 genetic variant has been associated with healthy ageing and exceptional longevity [19]. Notably, the genetic transfer of this variant into aged C57BL/6J mice revealed a deceleration in the progression of frailty, accompanied by improvements in several ageing biomarkers and multiple facets of health with a “rejuvenating effect” on the immune system [20]. The C57BL/6J strain, boasting a relatively abbreviated lifespan and a suite of ageing biomarkers, serves as an apt model for investigating therapies designed to extend healthy ageing and longevity [20]. Epigenetic clocks founded on DNA methylation profiles have emerged as dependable molecular markers of ageing. Simultaneously, frailty assessment tools provide a comprehensive evaluation of overall health during the ageing process [21]. In the study published in this S.I., Giuliani et al. [21] demonstrate that systemic gene transfer of LAV-BPIFB4 within aged C57BL/6J mice corresponded to a significant reduction in the epigenetic clock-based biological age, as evaluated using a three-CpG clock methodology. The authors showed that the gene therapy with LAV-BPIFB4 decelerated the age-related DNA hypomethylation in blood cells of old mice, reducing the epigenetic ticking rate, likely mainly in males. Moreover, the introduction of LAV-BPIFB4 gene transfer correlated to a reduction in the frailty index. These discoveries provide additional weight to the utilization of LAV-BPIFB4 gene therapy as a potential inducer of beneficial effects on the epigenetic mechanisms intricately linked with ageing and frailty in aged mice. The implications of these outcomes extend to potential therapeutic avenues aimed at preventing frailty in human populations. As our understanding of the molecular intricacies underlying longevity and healthspan deepens, these insights hold promise for advancing strategies that foster healthier ageing and enhance the quality of life in both laboratory models and, ultimately, humans.

The rejuvenating effect on the immune system alluded to by the authors brings forth another topic that has not been addressed in this S.I., namely, the potential role of the immune system in achieving longevity. This notion is not universally accepted, given that centenarians exhibit age-related immune system alterations [22,23]. However, findings from studies conducted on semi- and supercentenarians lend support to the idea that immune ageing might be more of a differential adaptation than a general immune alteration. From this perspective, the oldest centenarians have successfully adapted to a history of antigenic burden, thus attaining exceptional longevity, which can at least in part be attributed to the changes in their immune system [22,23,24,25].

## 5. Diet

The pursuit of longevity is not a recent human aspiration, but has been a longstanding endeavor for a considerable period. Throughout this enduring journey of uncovering effective means to elongate life, numerous avenues have garnered human enthusiasm. One such avenue is caloric restriction (CR), which was initially hailed as a universally beneficial approach for prolonging both lifespan and healthspan. But where does CR stand today, in light of advancing scientific insights? Does a sustained reduction in food consumption continue to hold the promise of unlocking the secrets to the long and vibrant lives of centenarians? Does the adage “eat less to live longer” still resonate? In this S.I., the comprehensive review of Dakic et al. [26] seeks to distil the present body of literature surrounding CR as a potential intervention to extend the human lifespan. Moreover, it delves into the complex metabolic pathways that underpin the observed effects. As our understanding of human biology deepens and our grasp of the factors governing ageing becomes more refined, the evaluation of CR’s potential as a life-extending strategy takes on a new dimension. Numerous studies discussed in the review highlight the correlation between human longevity and notable enhancements in glucose regulation, insulin sensitivity, and a reduction in plasma insulin growth factor-1 levels. The recognition that CR stands out as the most prominent positive influencer of these metabolic processes further underscores its potential to combat obesity, metabolic disorders, and foster healthier dietary habits in contemporary society, thereby potentially extending the lifespan. However, caution is warranted, as CR might entail adverse effects, rendering it an inadvisable approach for older individuals who face risks of undernutrition, sarcopenia, and are more prone to a normal weight rather than obesity. Instead of a simple reduction in calorie intake, a comprehensive longevity diet encompassing diverse nutritional facets emerges as a multi-pronged approach tailored to individual age and health status. In this light, a calorie reduction, integrated as a constituent of a holistic health-conscious dietary strategy, could constitute a valuable element of standard healthcare. It holds potential as a proactive anti-ageing measure, contributing to a broader framework for promoting well-being and longevity that accounts for the multifaceted nature of human health across the lifespan.

## 6. Conclusions

In alignment with the theme of our S.I., these five manuscripts have unveiled novel and promising discoveries that contribute significantly to our comprehension of the molecular underpinnings of longevity. This collection of papers holds particular significance, as the exploration of factors that lay the foundation for a long and healthy lifespan carries profound implications for the realm of translational medicine. These findings serve as a vital bridge between fundamental research and practical applications, fostering potential interventions and strategies aimed at enhancing human well-being and extending the years of healthy living.
